# Body adaptation to Dance: A Gerontological Perspective

**DOI:** 10.14336/AD.2020.1107

**Published:** 2021-06-01

**Authors:** Piotr Gronek, Michał Boraczyński, Aline Nogueira Haas, Jan Adamczyk, Mariola Pawlaczyk, Wojciech Czarny, Cain CT Clark, Urszula Czerniak, Anna Demuth, Roman Celka, Paulina Wycichowska, Joanna Gronek, Magdalena Król-Zielińska

**Affiliations:** ^1^Faculty of Sport Sciences, Poznan University of Physical Education, Poznan, Poland.; ^2^Department of Public Health, Faculty of Health Sciences, Collegium Medicum, University of Warmia and Mazury, Olsztyn, Poland.; ^3^School of Physical Education, Physiotherapy and Dance, Federal University of Rio Grande do Sul, Porto Alegre, Brazil.; ^4^Department of Geriatric Medicine and Gerontology, Poznan University of Medical Sciences, Poznan, Poland.; ^5^College of Medical Sciences, Institute of Physical Culture Studies, University of Rzeszow, Rzeszow, Poland.; ^6^Faculty of Health and Life Sciences, Coventry University, Coventry, England.; ^7^Department of Anthropology and Biometry, Faculty of Sport Science, Poznan University of Physical Education, Poznan, Poland.; ^8^Department of Physical Education and Lifelong Sports, Faculty of Sport Science, Poznan University of Physical Education, Poznan, Poland.

**Keywords:** Cognition, dance, motor control, older adults, physiological parameters

## Abstract

A number of studies have investigated the effectiveness of dance in older adults in the context of healthy aging. Analysing results across studies is important to understand whether dance in older adults is an effective adjunctive intervention for the healthy aging. To summarize the current research results about the effectiveness of dance in older adults in the context of healthy aging, and to identify key areas for future research. The search was conducted in Web of Science, PubMed and Google Scholar databases, using the following search string and Boolean logic (‘AND’, ‘OR’) locating studies published between database inception and September 2018: Dance OR contemporary dance OR ballroom dance OR Latin dance OR standard dance OR hip-hop dance OR tango AND Cardiovascular OR circulation AND Emotion OR well-being OR blood pressure OR disease OR thrombosis OR vascular OR glucose OR blood OR cardiac OR mental OR heart rate. Two reviewers independently extracted studies data. Eight suitable publications were included. The results showed that dance promote improvements in cognitive parameters when compared to other types of exercise or no-exercise. Significant effects were found on some physiological parameters, even after a short intervention period. Dance proved to be able to assist older adults in the context of healthy aging. The improvements in the cognitive, physiological and motor control parameters are very relevant for this population, due to the impact in a better quality of life.

During aging process, the pharmacological treatment in certain cases if necessary is indispensable and this fact is indisputable. However, any non-pharmacological procedures, interventions and programmes that does not disrupt correct bodily function regardless of age group but especially for the older adults is of interest, and even enthusiasm, provided it can be effective in the prevention of certain disease or simply improving well-being. This is especially true when positive effects are elicited on the cardiorespiratory fitness, psycho-motor skills [1], nervous system [2], vascular aging [3], cognition, mood, and quality of life [4], as an effect of regular non-pharmacological treatment.

One prominent example is dancing, which represents one of the best physical activity (PA) especially recommended for older adults (≥60 y) [5] and better than any fitness sport due to the possible widest impact spectrum to the body including physical (endurance, muscular strength, flexibility), psychological (cognition) and social (ensuring the need of closeness, reducing of loneliness) needs [6].

Despite the fact that exercise is recommended by the World Health Organization (WHO) to maintain health and prevent disease [7] and preserving healthy aging process age standardised prevalence of insufficient PA reached 27.5% of global population in 2016 [8]. Consequences of physical inactivity include among others obesity [9], cancer and coronary heart disease [10], sarcopenia [11], cerebrovascular disorders [12], circulatory diseases [13] and frailty [14]. On the other hand, PA can contribute to ameliorate such disorders as depression and anxiety [15], dementia and heart failure [16], stroke [17], cognition impairment [18] and sleep problems [19,20].

Physical inactivity is widely observed among individuals aged ≥ 60 years [21], although many older adults are aware of benefits toward PA [22]. Identified barriers include pre-existing medical conditions, physical limitations, time constraints, low self-efficacy, and culture restrictions [23].

It seemed essential to investigate interventions designed to promote quality of life (QOL) and health for adults over age 60 years, helping them to ameliorate deterioration related with aging [24, 25] since with the increase of life expectation, the proportion of people aged over 60 years is growing faster than any other age group in almost every country worldwide [26].

Different studies showed that PA could improve age-related changes and among different kind of PA dance is increasingly used as an intervention due to it combines many diverse features related to the physical and psychological aspects [25].

Dancing is a social activity that comprises rhythmic, motor coordination, balance and memory, apart from its requirements for PA. These characteristics make dance a potentially powerful intervention for older adults [27, 28]. However, still essential is adjustment of intensity of physical effort which depends on style/technique that may significantly differ from low aerobic workload (tango, waltz) applied below the aerobic threshold to high anaerobic intensity during performance of foxtrot, physical dance, break-dance among others. There is observed positive influence of PA on cardiorespiratory fitness, motor control, plasticity of brain network [29] and improvement in cognitive tasks [30] that has been of high priority and challenge for older adults and make dance highly interesting in supporting acting of ameliorate aging among individuals aged ≥60 years. Dance especially improves cognitive flexibility in aging [31], social inclusion for people with dementia and carers through sharing dance [32], significant decreases in loneliness, depression, and negative mood (d = 0.33 - 0.42, p < 0.05), improved daily functioning (d = 0.40, p < 0.01) and diurnal cortisol slope (d = 0.30, p < 0.01) among older adults with mild dementia [33].

A number of studies have investigated the effectiveness of dance in older adults in the context of healthy aging. Analysing results across studies is important to understand whether dance in older adults is an effective adjunctive treatment for the healthy aging. This review aimed to summarize the current research results on this topic, and to identify key areas for future research.

## MATERIALS AND METHODS

### Search strategy

The search for this narrative review was conducted in Web of Science, PubMed and Google Scholar databases, using the following search string and Boolean logic (‘AND’, ‘OR’) to locate studies published between database inception and September 2018: Dance OR contemporary dance OR ballroom dance OR Latin dance OR standard dance OR hip-hop dance OR tango AND Cardiovascular OR circulation AND Emotion OR well-being OR blood pressure OR disease OR thrombosis OR vascular OR glucose OR blood OR cardiac OR mental OR heart rate.

Coding of papers only allowed for studies that included at least one typology of dance, including but not limited to: contemporary, ballroom, Latin, standard, hip-hop, tango. The articles must refer to some cardiovascular or circulatory component; and must report a physiological or cognitive variable(s), in participants ≥50 years old. Studies of varying designs were acceptable for the purposes of this review however, technical reports, review articles, non-human based studies, or studies that did not report a physiological or cognitive output were not considered further.

### Study Characteristics

We conducted multiple searches in each selected databases and additional searches for relevant references and citations linked to the studies obtained during this primary search. The selection process sought to identify studies, of varying design, with no age restriction, that investigated multiple typologies of dance, cardiovascular and circulatory considerations and physiological and cognitive impact, and were published in English between database inception and September 2018. Two reviewers assessed all titles and abstracts and all full-text articles; decisions to accept or reject a paper were agreed between the first and second reviewer, and a third, independent reviewer helped achieve consensus if the first and second could not agree.[Fig F1-ad-12-3-902]

**Figure 1. F1-ad-12-3-902:**
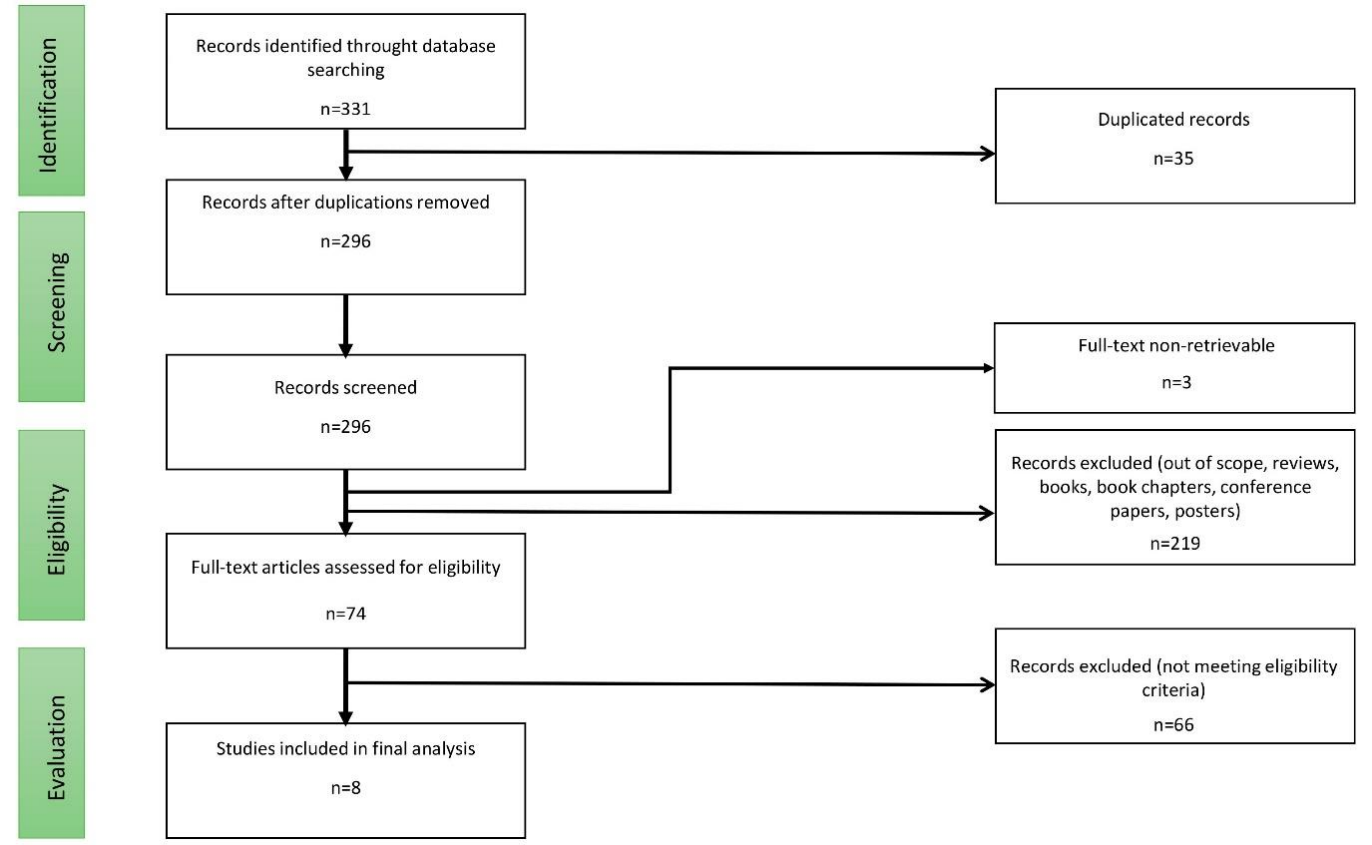
Flow chart of study selection procedures.

## RESULTS

Eight studies were included, which were in accordance to the eligibility criteria of this research and presented the best quality. The included studies were subdivided into three topics: 1) cognition, 2) physiological aspects, 3) motor control.[Table T1-ad-12-3-902]

**Table 1 T1-ad-12-3-902:** summarizes the included articles.

	Study	Aim	Population	Measurement	Baseline	Follow-up	Conclusion
1	Coubard et al. (2011)	The impact of contemporary dance (CD) improvisation on attentional control of older adults, as compared to two other motor training programs, fall prevention and Tai Chi Chuan	Dance: N=16 (all F); 73.6±5.4y. Fall: N=67 (3 M); 74.7±6.7y. TaiChi: N=27 (3 M); 71.5±7.4y.	AWP low accuracy (n/1)	Dance: 0.87±0.09 Fall: 0.82±0.04 TaiChi: 0.89±0.07	Dance: 0.69±0.10 Fall: 0.78±0.05 TaiChi: 0.88±0.08	Dance improved switching but not setting or suppressing cognitive attention.
				AWP high accuracy (n/1)	Dance: 0.37±0.12 Fall: 0.39±0.06 TaiChi: 0.26±0.09	Dance: 0.31±0.12 Fall: 0.45±0.06 TaiChi: 0.41±0.10	
				Stroop interference ratio (n/1)	Dance: 0.46±0.03 Fall: 0.50±0.01 TaiChi: 0.52±0.02	Dance: 0.48±0.03 Fall: 0.49±0.01 TaiChi: 0.51±0.02	
				Stroop interference rate (%)	Dance: 3.29±1.79 Fall: 2.10±0.88 TaiChi: 2.34±1.38	Dance: 6.65±2.57 Fall: 1.98±1.25 TaiChi: 2.41±1.98	
				Rule shift (n/4)	Dance: 2.19±0.28 Fall: 3.03±0.13 TaiChi: 3.15±0.21	Dance: 2.69±0.29* Fall: 2.97±0.14 TaiChi: 3.26±0.22	
				Rule shift switch ER (%)	Dance: 43.7±7.50 Fall: 19.4±3.67 TaiChi: 16.0±5.77	Dance: 27.1±8.01* Fall: 21.2±3.91 TaiChi: 15.6±6.17	
2	Kattentsroth et al. (2013)	The effects of a 6-month dance class (1h/week) on a group of healthy older individuals compared to a matched control group (CG) on cognition and motor control	Dance IG (n = 25, 17 women; age, 68.60 ± 1.45 years); non- Dance CG (n = 10, 7 women; age, 72.30 ± 1.84 years)	Cognition	Dance: 0.64 ± 0.02 Control: 0.63 ± 0.04	Dance: 0.72 ± 0.02* Control: 0.63 ± 0.04	Dance improves motor control and cognition, but not cardiorespiratory fitness
				Motor control	Dance: 0.73 ± 0.01 Control: 0.73 ± 0.02	Dance: 0.78 ± 0.01* Control: 0.72 ± 0.02	
				Cardio-respiratory fitness: spiro-ergometry	Dance: absent values Control: absent values	Dance: no change, absent values Control: no change, absent values	
3	Rodrigues-Krause et al. (2018)	To describe cardiorespiratory responses of a dance session for older women, and to identify intensity zones in relation to peak oxygen consumption (VO_2_peak), first and second ventilatory thresholds (VT1 and VT2).	Ten women from 60 to 75 years old, body mass index (BMI) lower than 35 kg/m^2^	Oxygen uptake (VO_2_)	N/A	VO_2peak_: 23.3 ± 4.3 VT1: 17.2 ± 3.5 Dance: 16.1 ± 3.3	Cardiorespiratory demands of a dance class for older women are at low aerobic intensity. Show was similar to VT1, indicating that a dance class may be modulated to improve aerobic fitness, at least at initial stages of training
4	Rossmeissl et al. (2016)	To assess the feasibility of a 12-week ZumBeat dance intervention in sedentary, postmenopausal overweight women	N=17 (all F); 55±6y; 167±9cm; 85±6.5 kg	VO_2peak_	24.3 ± 2.9	23.9 ± 3.2	A 12-week ZumBeat dance intervention may not suffice to Increase cardiorespiratory fitness in postmenopausal overweight women.
				Blood pressure	SBP: 126.6 ± 14.2 DBP: 80.4 ±9.9	SBP: 131.0 ± 12.3 DBP: 83.3 ±9.8	
				Quality of life	79.4 (17.2)	88.7 (8.8)*	
5	Sampaio et al. (2016)	To evaluate cardiac autonomic modulation in individuals with chronic stroke post-training using a virtual reality-based aerobic dance training paradigm	Eleven (6 F) community-dwelling individuals with hemiparetic stroke; 61.7 ± 4.3 years; 93.48 ± 41.27 kg; 169.27; ± 8.80 cm.	High-frequency (HF) power (cardiac parasympathetic activity)	51.5± 19	59.7 ± 8*	Virtual-reality dance can be used to improve cardiac autonomic control
				low-frequency (LF) power (parasympathetic-sympathetic balance)	48.4 ± 20.1	40.3 ± 8.0*	
				LF/HF (sympatho-vagal balance)	1.6 ± 1.9	0.8 ± 0.26*	
6	Kim et al. (2011)	To examine the effects of dance exercise on cognitive function in older patients with metabolic syndrome	Dance (N=26, 19 F): 68.19 ±3.66y Control (N=12, 10 F): 68.16 ±5.14y	BMI (kg·m-2)	Dance: 25.71 (2.87) Control: 25.90 (2.38)	Dance: 25.55 (2.95) Control: 25.15 (2.19)	Dance exercise may reduce the risk for cognitive disorders in older adults, but has a limited effect on physiological health markers.
				WC (cm)	Dance: 94.43 (6.22) Control: 92.95 (5.05)	Dance: 90.27 (6.13) Control: 88.28 (2.01)	
				Triglyceride (mg·dl-1)	Dance: 126.76 (54.41) Control: 134.90 (50.0)	Dance: 120.21 (45.68) Control: 124.78 (40.54)	
				Glucose (mg·dl-1)	Dance: 102.00 (10.86) Control: 96.55 (6.71)	Dance: 110.17 (13.89) Control: 107.00 (6.00)	
				HDL cholesterol (mg·dl-1)	Dance: 53.38 (14.20) Control: 44.08 (7.57)	Dance: 53.29 (14.19) Control: 46.11 (7.74)	
				SBP (mmHg)	Dance: 131.92 (11.73) Control: 133.41 (7.68)	Dance: 123.41 (10.83) Control: 130.71 (8.61)	
				DBP (mmHg)	Dance: 80.11 (6.95) Control: 80.91 (5.83)	Dance: 74.00 (8.04) Control: 80.15 (6.93)	
				Cognition	Dance: 68.42 (9.75) Control: 60.66 (13.25)	Dance: 75.25 (9.23) * Control: 63.66 (12.58)	
7	Serrano-Guzman et al. (2016)	To test the effectiveness of a dance therapy program in postmenopausal women	Dance (N=27, all F); 69.07±4.41y; 28.64±3.69 Control (N=25, all F); 69.48±3.22y; 29.31±3.69	Timed up and go (TUG)	Dance: 10.08_2.41 Control: 10.36_2.20	Dance: 8.29_1.39* Control: 10.44_2.09	Spanish dance therapy may be effective to improve mobility, balance, and levels of physical activity and fitness in sedentary postmenopausal women
				Cardiorespiratory fitness (1-5 points scale)	Dance: 1.40_0.50 Control: 1.24_0.43	Dance: 2.14_0.45* Control: 1.16_0.55	
				SBP (mmHg)	Dance: 119.4_13.18 Control: 123.2_11.44	Dance: 117.2_10.94 Control: 125.0_9.32	
				DBP (mmHg)	Dance: 68.33_8.32 Control: 70.40_8.77	Dance: 67.59_7.64 Control: 74.08_7.14	
				TUG cognitive (s)	Dance: 11.32_3.57 Control: 13.00_8.93	Dance: 9.89_2.29* Control: 11.71_3.16	
8	Wu et al. (2016)	To assess the effect of low-impact dance in older sedentary women	Dance = 60 ± 4 years, Control = 58 ± 5 years	HDL (mg·dl-1)	Dance: 53.5 ± 9.1 Control: 54.9 ± 7.6	Dance: 58.8 ± 6.3* Control: 54.0 ± 7.3	In addition to improvements in blood lipids and body fat percentages, a low impact dance program increases capability in performing common daily activities in older women.
				LDL (mg·dl-1)	Dance: 138.5 ± 15.6 Control: 138.2 ± 16.1	Dance: 131.2 ± 16.2* Control: 135.6 ± 13.1	
				BF%	Dance: 32.4 ± 6.5 Control: 32.7 ± 5.6	Dance: 30.0 ± 6.0* Control: 33.0 ± 5.2	
				Knee torque (Nm)	Dance: 34.2 ± 2.9 Control: 34.0 ± 1.9	Dance: 37.0 ± 2.5* Control: 35.4 ± 1.8	

Abbreviations: AWP low/high - arithmetic word problems with low/high planning demand; BMI - body mass index; DBP - diastolic blood pressure; HDL - high-density lipoprotein ; LDL - low-density lipoprotein; LF - low-frequency; HF - high-frequency; TUG - Timed up and go; SBP - systolic blood pressure; VO_2max_ - maximal oxygen uptake; VO_2peak_ - peak oxygen uptake; WC - waist circumference.

## Cognition

Coubard *et al*. [31] examined the impact of contemporary dance improvisation on attentional control of 16 older adults (mean age 73.6±5.4), as compared to two other motor training programs, fall prevention (67 participants, mean age 74.7±6.7) and Tai Chi Chuan (27 participants, mean age 71.5±7.4). All participants performed pencil-and-paper tests (Arithmetic word problems, Stroop test and Rule shift cards test) before and after 5.7-month training, to measure setting, suppressing, and switching attention tasks. Results indicated that contemporary dance improved switching attention or cognitive flexibility, but not setting or suppressing attention. In contrast, neither fall prevention or Tai Chi Chuan showed any effect. The authors suggested that contemporary dance improvisation maybe a useful way to boost cognitive flexibility, working as a training to change, inducing plasticity in flexibility attention.

Kattenstroth *et al*. [28] investigated the effects of a 6 months dance class (1 h/week) in cognition on a group of 25 healthy older individuals (mean age 68.60±1.45) compared to 10 healthy older individuals in the control group (mean age 72.3±1.84) that continues their usual lifestyle. Cognitive performance and neuropsychological status were assessed using the Repeatable Batter of Neuropsychological Status (RBANS). After 6 months of dance intervention, dance group improved their cognition domain (p ≤ 0.001) while the control group presented no changes. The authors concluded that dance could help to ameliorate a wide spectrum of age-related decline.

In the prospective pilot study, Kim *et al*. [34] analysed the effects of dance exercise on cognitive function in 38 participants aged over 60 years, with metabolic syndrome and normal cognitive function (26 dance group and 12 control group). The dance group performed Cha-cha-cha, Latin dance style twice a week for 6 months. Cognitive function was assessed using the Korean version of the Consortium to Establish a Registry for Alzheimer’s disease (CERAD-K). Compared with the control group, the dance group significantly improved in verbal fluency (p = 0.048), word list delayed recall (p = 0.038), word list recognition (p = 0.007), and total CERAD-K score (p = 0.037). The authors concluded that six months of dance exercise improved cognitive function and may reduce the risk of cognitive disorders in older adults with metabolic syndrome.

Serrano-Guzman *et al*. [35] tested the effectiveness of a dance therapy program in dual-tasking performed during the test Timed up and Go (TUG). Fifty-two sedentary postmenopausal women (mean age 69.27±3.85 years) that were randomly assigned to receive either dance therapy (n=27) or self-care treatment advice (n=25). The intervention group participated in dance therapy classes based on Spanish folk dance (Flamenco and Sevillanas), 3 sessions weekly, for 2 months. The control group was provided a booklet containing PA recommendations. The intervention group showed significant improvements in cognitive TUG (p=0.029) and the authors concluded that Spanish dance therapy may be effective to improve mobility and balance, especially in cognitive dual-tasking.

## Physiological aspects

Kattenstroth *et al*. [28] tested changes in the level of aerobic fitness among 35 healthy older adults (range 60-94 years) by spiroergometry, assessing VO_2peak_ (L/min). Twenty-five older adults were attended the dance classes called Agilando for 1h/week for 24 weeks (intervention group), and 10 were not (control group). In neither of the groups, significant changes in cardio-respiratory performance were observed.

Rossmeissl *et al*. [36] assessed the feasibility and effect of a 12-week ZumBeat (Zumba-style) dance intervention on cardiorespiratory fitness and psychosocial health among 13 postmenopausal women 45 and 65 years of age. Peak oxygen consumption (VO_2peak_) and maximum heart rate (HR_max_) were assessed during spiroergometric cardiopulmonary exercise testing on a treadmill. Before the dance training the mean results of VO_2peak_ (mL⋅kg^-1^⋅min^-1^) and HR_max_ (bpm) were 24.3 ± 2.9 and 161.8 ± 15.5, and after the intervention 23.9 ± 3.2 and 164.5 ± 17.0, respectively. Neither VO_2peak_ nor HR_max_ parameter changes were significant.

Sampaio *et al*. [37] evaluated cardiac autonomic modulation in individuals with chronic stroke post-training using virtual reality-based aerobic dance training paradigm. Eleven community-dwelling individuals with hemiparetic stroke (61.7 ± 4.3 years) received a virtual reality-based dance paradigm for 6 weeks using the commercially available Kinect dance video game “Just Dance 3”. There were 20 training sessions, each lasted about 1.5 hour. Heart rate variability (HRV) data during supine and standing pre- and post-intervention resulted in a significant alteration in HRV in the supine position post-intervention (p<0.05). Participants also showed significant gains in VO_2max_ (p<0.05) suggesting a significant increase in aerobic capacity post-intervention in comparison to pre-intervention. Authors described physical activity measure as the number of steps taken in each session: there was a significant increase in the number of steps recorded between the first and last training session (F_(2, 20)_ = 29.342, p<0.01). The number of steps increased from 761.4 ± 401.82 on the first session to 1679.9 ± 496.98 steps recorded at the tenth session.

Serrano-Guzman *et al*. [35] evaluated the effectiveness of a dance therapy program in improving mobility, balance, body mass, quality of life and blood pressure. The group of 52 white postmenopausal women with at least 12 months of amenorrhea and prehypertension or hypertension were allocated to either dance therapy group or self-care advice group. Dance classes contained 24 sessions, three times per week, for 2 months. Each session lasted 50 minutes. Mobility was assessed using various types of TUG test (in standard conditions, with both manual and cognitive task) and One Leg Stance test. For majority of above tests the significant changes between groups were observed (group × time interaction). Only TUG with manual task showed no significant differences. There were also no significant changes of blood pressure.

Wu *et al*. [38] analysed effect of Low-Impact Dance on the joint range of motion of lower extremities and knee extension torque. A total of 32 healthy volunteer female participants were randomly assigned to either the low-impact dance (LOD) or sedentary (SED) group. The intervention was dance program consisted of three classes per week on non-consecutive days for a total of 16 weeks. Each session lasted about 60 minutes. For knee extension, significant interactions were found in the dominant and nondominant legs (p <0.05), for which the postintervention value for the LOD group was significantly higher than its preintervention value (p=0.031) and the SED group’s value at the same time point (postintervention). For ankle inversion, significant interactions were also found in the dominant and nondominant legs (p<0.05) and the LOD group was significantly higher than the SED group in this variable after the intervention (p<0.05). However, ankle eversion of the dominant leg was significantly higher in the LOD than in the SED group after intervention with the low-impact dance program. Ankle dorsiflexion showed significant interactions in the dominant and nondominant leg (p<0.05), with the LOD group significantly higher than the SED group after intervention. However, for ankle plantar flexion, there were no significant differences in the dominant or nondominant leg from pre- to postintervention (p>0.05). For knee extension torque, there was no significant difference in the dominant or nondominant leg between the LOD and SED groups before the intervention (p>0.05). After the intervention, neither the dominant nor nondominant leg for the values in the SED group differed significantly compared with the values at preintervention (p > 0.05). However, there were significant interactions for this variable of dominant and nondominant leg in the LOD group, which showed significant increases compared with the same group preintervention and the SED group postintervention (p<0.05 for both the dominant and nondominant leg), respectively.

Rodrigues-Krause *et al*. [39] assessed cardio-respiratory responses of a dance session for older women and identified intensity zones in relation to peak oxygen consumption (VO_2peak_), first and second ventilatory thresholds (VT1 and VT2) among ten women from 60 to 75 years old. The results of VO_2_ (mL⋅kg^-1^⋅min^-1^) are: VO_2peak_ (23.3 ± 4.3), VT1 (17.2 ± 3.5) and VT2 (20.9 ± 3.4). Dancing: warm-up (12.8 ± 2.4, ~55%VO_2peak_), across-the-floor (14.2 ± 2.4 ~62%VO_2peak_), choreography (14.6 ± 3.2 ~63%VO_2peak_) and show (16.1 ± 3.3, ~69%VO_2peak_). Since cardiorespiratory demands of a dance class for older women are at low aerobic intensity, authors concluded that a dance class may be modulated to improve aerobic fitness, at least at initial stages of training.

## Motor control

Kattenstroth *et al*. [28] performed a comprehensive assessment covering reaction time, mobility, tactile, and postural performance in order to explore the potential beneficial effects of a 6-month dance intervention in 2 groups of older adults participants having no regular record of dancing or sporting activities for at least the previous 5 years.

Choice reaction and visual analysis of processing time were performed using the standardized Reaction Time Analysis (RA) implemented in the Viennese test system (Dr. G. Schuhfried GmbH, Mödling, Austria), which is based on a model assuming additivity of factors of the perception, cognitive processing and motor response. Posture and balance were analysed using a force platform. The performance of small hand and arm movements was evaluated using a commercial, computer-based test-battery for clinical neuropsychological research. This test assesses the degree of ataxia and the speed of movement by the ability of making rapid repeated aimed movements. Touch threshold was evaluated by probing the fingertips with von Frey filaments (Marstocknervtest, Marburg, Germany). Spatial 2-point discrimination (2pd) thresholds were assessed on the tips of the left (LID) and right (RID) index fingers by using the method of constant stimuli.

In all tests contributing to reaction times, significant improvements were found for the dance group while no differences were found in the control group. In the domain Hand/Motor performance, subjects in the dance group showed a significant reduction of the number of errors for steadiness and a speeding up for aiming and pin plugging (Hand/motor performance for both groups before and after the class: IG pre 0.73 ± 0.01 - post: 0.78 ± 0.01 and CG pre 0.73 ± 0.02 - post 0.72 ± 0.02; *p*-value 0.073). For the domain of tactile performance, subjects in the dance group showed lower touch and 2pd thresholds, made fewer mistakes, and were faster in the haptic object recognition task. Postural performance improved significantly among subjects in the dance group. No differences were ascertained for subjects in the control group.

Further analysis of individual performance by linear correlation analysis between the individual performance at baseline (prior to the intervention) and gain in performance after the intervention showed that subjects characterized by a low performance at the baseline attained higher individual gains. Significant correlations were found for reaction times (r = -0.449, p = 0.032), hand/motor performance (r = -0.632, p = 0.002), the tactile domain (r = -0.692, p ≤ 0.001), and posture (r = -0.787, p ≤ 0.001).

## DISCUSSION

This review aimed to summarize the current research on the effectiveness of dance in older adults in the context of healthy aging and to identify research gaps that need to be addressed in the future.

In total, we found 8 suitable publications that investigated the specific effects of dance in older adults on cognitive, physiological, and motor control parameters related with aging. The small number of included publications on this review, as well as the fact that the oldest publication was published in 2011, indicates that dance for older adults as a potential intervention to enhance healthy aging is a relatively new area of research. However, various outcomes have been investigated as cognitive, physiological, kinaesthetic, motor and non-motor symptoms.

It seems to be obvious that not all symptoms of aging can be reduced in the same way with different kinaesthetic, physiotherapeutic and physical activity approaches. However, this review, after analysed results across studies, aimed to indicate that these aging symptoms might be reduced due to participation in dance interventions.

Furthermore, the study results showed especially promoting aspects of dance intervention in cognitive parameters what seems to be interesting in context of WHO principles and modern approach there is “no health without mental health” [40]. Since a major goal of modern medicine is to preserve quality of life this translates into concept of healthy aging, which is “considered to comprise avoiding disease and disability, maintaining good cognitive and physical function, and remaining actively engaged in life” [41]. Can be added as follows - with avoiding medicine treatment where possible. The notion of a good life can be observed from subjective to the objective, where this spectrum incorporates a number of existing qualities of life theories. Ventegodt et al. [42] call this spectrum the integrative quality-of-life (IQOL) theory and this is the theoretical aspect assumed in this study. A recent systematic review provided substantial evidence that music and dance activities enhance participants’ physical, cognitive, and social determinants of health (such as stress, social isolation, autonomy) and individual wellbeing across all groups, and interventions can be highly effective and able to be adequately maintained [43]. Most of the studies in this review showed significant improvements in cognitive function, revealed significant overall effects in favour of dance, which are moderate for a useful way to boost cognitive function, and may reduce the risk for cognitive disorders in older adults, especially with metabolic syndrome [34]. Dance is effective as well to improve mobility and balance especially in cognitive dual-tasking [35].

Cognition is a general term that refers to the mental abilities, which includes the short- and long-term memory, working memory, verbal fluency, and the ability to feel, to think, to reason, to form complex structures of thought and to respond to external stimuli [44, 45]. Cognitive losses in older adults can cause disorganized thinking, decreased of cognitive flexibility, bradyphrenia (slowness of thought), loss of recent memory and difficulties in planning, in focusing and in attention. Cognitive decline can generate problems in social life and in daily life activities on this population. Improvement of the cognitive function or reduction of the risk of cognitive disorders in older adults it is an important result for this population, since older adults lead to difficulties in cognitive aspects. A systematic review introduced by Predovan *et al*. [46] outlined some of the possible mechanisms by which dance could positively impact cognition in older adults (429 participants [70% women], with a mean age of 73.2 years old). The authors highlighted three specific mechanisms: (1) the complexity of coordination learning during the act of dancing, (2) effect on depression symptoms (by evoking emotional experiences and physiological reactions), and (3) improvement of cognitive performance via the presence of music during physical exercise. The applicative information they also found was that dance interventions, lasting between 10 weeks and 18 months, were related to either the maintenance or improvement of cognitive performance.

In the cardiorespiratory parameters we found different results. Rodrigues-Krause *et al*. [39] indicated that dance could help to ameliorate a wide spectrum of age-related decline contributing to a healthy aging, including increases in VO_2peak_, lower body muscle power and static balance. Interestingly, we have observed non-significant changes in blood pressure [35], VO_2peak_ and HR_max_ [36]. Kattenstroth *et al*. [28] found no significant effects in the cardio-respiratory parameters after a long duration dance intervention (six months).

To create positive changes in cardiorespiratory system, increasing aerobic capacity, aerobic endurance, maximal oxygen uptake and other physiological parameters, there is needed appropriate adjustment to age and health status bout of appropriate intensity, volume and weekly frequency of exercise. During aging aerobic and anaerobic capacity and power decline [47-49]. However, in an opposite tendency it is possible to maintain. Many studies have focused on physical exercise intervention programs aimed at improving cardiorespiratory function measured by aerobic endurance in older adults [50-54].

It is particularly interesting that significant effects were found on some physiological parameters, as VO_2max_ [39] and mobility and balance especially in cognitive dual-tasking [35], even after a short intervention period over eight weeks of training. Interestingly, older adults are interested to get feedback about the procedures more intensively than any other age groups. Additionally, adults over 65 years old have tendency to measure what is only possible and available to compare eventual differences between sessions. The fact that symptoms may improve even after a relatively short period of intervention appears as a high motivational factor for older adults to participate in such kind of intervention. However, more of the analysed treatments showed positive results in relatively long-lasting training period, duration at least 5 months [28, 31, 34].

For older adults, it is also possible to observe training adaptation in terms of cardiorespiratory parameters. However, in their case, physiological processes look much different than young people. Among others, in older adults appears a natural decrease in aerobic and anaerobic capacity resulting from the reduction of the body's tolerance to training, lowering the maximum values of the oxygen uptake, which reduces the maximal cardiac output (CO_max_). In addition, muscle mass plays a particularly important role in the elderly since muscle metabolism, and the associated myocyte oxygen, stimulates the cardiovascular system to produce more intense functioning. In the group of older adults, this is one of the main reasons for the decrease in the exercise capacity, especially after exceeding 60 years old and in women in the postmenopausal period.

Therefore, while slimming a figure resulting from lowering the level of adipose tissue is beneficial at any age, reduction of body weight resulting from the loss of muscle mass is not beneficial especially for older people. In summary, body weight reduction as the result of intensive endurance training and as the consequence increasing VO_2max_ in a young person is beneficial; however, in a group of older people this phenomenon to create quite the opposite result. Then, it is recommended to include resistance training for elderly, especially in people with sarcopenia and osteoporosis where the beneficial effect of resistance exercises is observed. On this way, dance interventions may not be sufficient if they are not accompanied by adequate resistance training. This view is in line with one of the recent meta-analyses carried out by Northey *et al*., [55] who concluded that an exercise programme with components of both aerobic and resistance-type training, of at least moderate intensity and at least 45 min per session (on as many days of the week as possible), is beneficial to cognitive function in adults aged >50 years.

The cardiorespiratory parameters look different in physically inactive people comparing to well-trained people, as improving cardiovascular parameters will naturally be more difficult for them. Kattenstroth *et al*. [28] observed the lack of further progressive decline in cardiorespiratory parameters in older adults, recognizing this as a positive phenomenon. There is no procedure at this age that could cause an absolute spectacular increase in cardiorespiratory parameters since for each decade cardiorespiratory functions naturally decline 2% per decade.

The main patients’ and therapists’ expectation are to receive beneficial and apparent effects in the shortest possible time. The burning issue is that long lasting treatment is not desirable and enough attractive for a patient, and especially adults over age 65 yrs.

The results of this narrative review need to be considered in light of the purpose and inclusion/exclusion criteria. The limitation of this study is we identified a relatively few research data examining the health and health-related physical fitness outcomes for older participants engaged in dance activities. However, major strengths of this study are (1) it provides up-to-date summary of dance studies in relation to cognitive and physical health status in older adults, (2) our inclusion and exclusion criteria did not negate studies with symptomatic samples and this potentially prevented the results bias. Although this review provides some evidence of the beneficial effects of regular dance activities on cognitive functions, physiological markers and multiple physical fitness components (mainly cardiorespiratory fitness), future studies are needed to validate the hypothesis that dancing could simultaneously improve cognition functions, physiological status and physical fitness of elderly populations. Moreover, future research should also move beyond investigating effectiveness and begin to refine the prescription of training to promote the greatest benefits in terms of healthy aging concept.

## Conclusion

Dance proved to be able to assist older adults in the context of healthy aging. The improvements in the cognitive, physiological and motor control parameters are very relevant for this population, due to the impact in better quality of life.

We identified a lack of studies and scientific information in the area of dance and older adults, indicating that this area of research is relatively new. It is suggested that another research conducted in this field, should include different outcomes and should be studied with greater and better scientific accuracy, as clinical trials.
